# Chronic Kappa opioid receptor activation modulates NR2B: Implication in treatment resistant depression

**DOI:** 10.1038/srep33401

**Published:** 2016-09-16

**Authors:** Shalini Dogra, Ajeet Kumar, Deepmala Umrao, Amogh A. Sahasrabuddhe, Prem N. Yadav

**Affiliations:** 1 Division of Pharmacology, CSIR-Central Drug Research Institute, Lucknow, UP 226031, India; 2 Division of molecular and structural biology, CSIR-Central Drug Research Institute, Lucknow, UP 226031, India

## Abstract

Psychotomimetic and prodepressive effect by kappa opioid receptor (KOR) activation in rodents and human is widely known. Significantly, recent clinical investigations demonstrated the salutary effects of KOR antagonists in patients with treatment resistant depression, indicating essential role of KOR signaling in refractory depression. This study was undertaken to reveal the molecular determinant of KOR mediated depression and antidepressant response of KOR antagonist. We observed that chronic KOR activation by U50488, a selective KOR agonist, significantly increased depression like symptoms (behavioral despair, anhedonia and sociability) in C57BL/6J mice, which were blocked by KOR antagonist norBNI and antidepressant imipramine, but not by fluoxetine or citalopram. Further, chronic KOR activation increased phosphorylation of NR2B subunit of NMDA at tyrosine 1472 (pNR2B NMDA) in the hippocampus, but not in the cortex. Similar to behavioral effects norBNI and imipramine, but not SSRIs, blocked NR2B phosphorylation. Moreover, KOR induced depression like behaviors were reversed by NR2B selective inhibitor Ro 25-6981. Mechanistic studies in primary cultured neurons and brain tissues using genetic and pharmacological approaches revealed that stimulation of KOR modulates several molecular correlates of depression. Thus, these findings elucidate molecular mechanism of KOR signaling in treatment resistant depression like behaviors in mice.

Depression is a complex and heterogeneous disorder that affects millions of people worldwide. Understanding the underlying mechanisms of a highly complex disease like depression is still one of the primary challenges for modern psychiatry. Over the last four decades, the prevailing hypothesis of depression has been the monoamine hypothesis which included the catecholamine^1^ and serotonin (5-HT) hypotheses^2^. The monoamine hypothesis originated from the mechanistic studies of the serendipitously discovered tricyclic antidepressants (TCAs) and monoamine oxidase inhibitors. The selective 5-HT reuptake inhibitor (SSRI), and 5-HT and norepinephrine (NE) reuptake inhibitor (SNRI) antidepressants that were launched during the 1980s and 1990s are still the first line treatment for depressive disorders worldwide. In general, SSRIs are better tolerated than most other types of antidepressants; therefore these are the first choice of medication for patients with major depression^3^. Even though there are multiple Food and Drug Administration approved SSRIs in the market, significantly large segment of patients with depression exhibit resistance to this class of drugs^4^. Although several rodent models of depression have been employed during last many decades, which either mostly uses various types and degree of stressors, or strains which are predisposed to depressive behavior, none of these have been clearly validated for the treatment resistant depression like phenotype^5^,^6^. Thus, there is an urgent need for a better model with clear underlying mechanism for the development of novel antidepressants for refractory depression.

Multiple lines of evidence suggests that glutamatergic neurotransmission mediated via N-methyl-D-aspartate receptors (NMDARs) play fundamental role in the pathophysiology of psychiatric disorders, including major depression and bipolar depression^7^,^8^. Drugs targeting NMDARs for the treatment of major depression have lately gained significant attention as they exhibit beneficial results in animal models as well as in depressed patients^9^,^10^. Particularly, ketamine exerts fast and robust antidepressant effects in the treatment-resistant depressed patients, whereas conventional antidepressants take several weeks for the therapeutic onset^11^. However, antidepressant effects of ketamine have been found to be short-lived^12^ and psychotomimetic properties associated with pan NMDA antagonism have been a major concern for long term clinical use of ketamine.

Opioid receptors are well known to regulate motivational processes and are recognized as important players in psychiatric illnesses that are due to reward dysfunction, such as drug addiction and depression^13^,^14^. Kappa opioid receptor (KOR) has been implicated in the behavioral consequences of stress, such as drug seeking and depression^14^,^15^. Notably, almost all KOR agonists exhibit dysphoric and psychotomimetic properties^16^,^17^, and KOR antagonists exhibit antidepressant effects in human and rodents^18^,^19^. Interestingly, Wistar Kyoto (WKY) rats - a putative genetic model of comorbid depression and anxiety, exhibit increased KOR expression in locus coeruleus^20^, decreased expression of BDNF in prefrontal cortex (PFC) and hippocampus^21^, and are resistant to SSRIs^22^. Although, these studies suggest that increased KOR activation might be a reason for resistance to SSRIs efficacy, the underlying mechanism(s) is not known, yet.

The present study was undertaken to reveal the molecular determinant of treatment resistant depression and to further elucidate the neurobiological predictor of antidepressant response. We demonstrated that persistent KOR activation by chronic treatment with U50488, a selective KOR agonist, increased depression like symptoms in C57BL/6J mice, which were blocked by KOR antagonist *nor*BNI and tricyclic antidepressant imipramine, but not by SSRIs fluoxetine and citalopram. Further, we showed that KOR activation leads to significant and selective phosphorylation of NR2B (pY1472) subunit of NMDAR complex in the hippocampus that seems to be associated with behavioral response of antidepressants. Importantly, depression like behaviour induced by KOR activation was reversed by NR2B selective inhibitor Ro 25-6981, signifying the role of KOR-NMDA cross talk in treatment resistant depression.

## Results

### Chronic KOR activation causes treatment resistant depression like symptoms in mice

**Figure 1:**
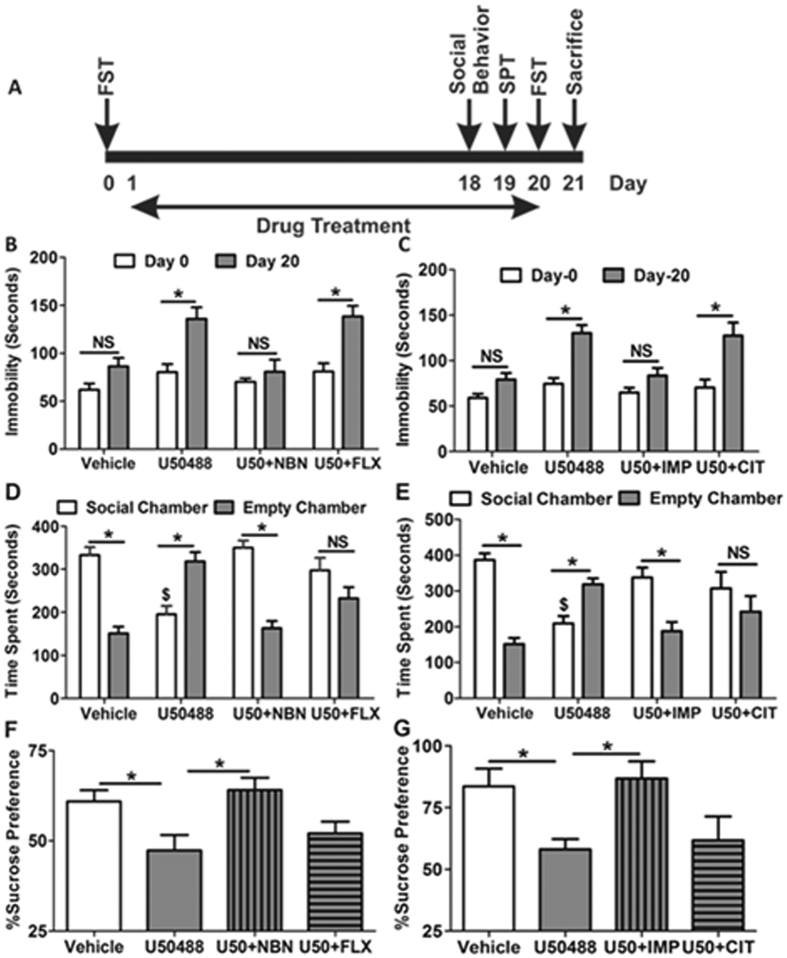
Chronic U50488 treatment induces depression like symptoms in mice. (**A**) A graphical representation of the experimental design. **(B,C)** U50488 (5 mg/kg; *i.p*.) treatment increased immobility time in the forced swim test on day-20 as compared to day-0. Treatment of KOR selective antagonist norBNI (10 mg/kg, two injection- day 0 and day 10, *i.p*.) blocked the U50488 induced increased immobility in FST, but fluoxetine (10 mg/kg; *i.p*.) co-treatment with U50488 for 20 days did not affect the immobility time. Co-treatment of imipramine (10 mg/kg; *i.p.*), but not citalopram (10 mg/kg; *i.p.*), with U50488 significantly blocked the effects of U50488 in FST. Each histogram represents mean ± SEM of 12–15 mice/group, *p < 0.0001 by two-way ANOVA followed by Bonferroni *post hoc* analysis for multiple comparisons. (**D,E**) U50488 treated animals exhibited marked social deficit by spending significantly more time in the empty chamber as compared to vehicle treated animals, which is blocked by norBNI or imipramine, but not by fluoxetine or citalopram. Each histogram represents mean ± SEM of 10–12 mice/group, *p < 0.0001 by one-way ANOVA followed by Newman-Keuls *post hoc* analysis for multiple comparisons. (**F,G**) U50488 treated animal exhibited marked reduction in sucrose preference, which is blocked by co-treatment with norBNI or imipramine, but not with fluoxetine or citalopram. Each histogram represents mean ± SEM of 6–7 mice/group, *p < 0.05 by two-way ANOVA followed by Newman-Keuls *post hoc* analysis for multiple comparisons. U50 = U50488, NBN = norBNI, FLX = Fluoxetine, IMP = Imipramine, CIT = Citalopram.

To determine the effect of sustained KOR activation on depression like behaviours in mice, C7BL/6J animals were treated for 20 days with either saline (*i.p.,* once daily), or analgesic dose (5 mg/kg) of a selective KOR agonist U50488 (Supplementary Fig. S1A,B; once, daily). Since higher doses (20–30 mg/kg) of KOR agonist U50488 have been shown to produce tolerance^23^, we chose lower but effective dose of U50488 (5 mg/kg) to avoid development of tolerance, but activate receptor persistently. Our results demonstrate that persistent KOR activation significantly increased behavioral despair like symptoms (measured by immobility time in forced swim test) in the mice (*F*_1,75_ = 31.04, *p* < 0.0001; two-way ANOVA), while the mice treated with vehicle for 20 days did not exhibit significant increase in this test. Further, U50488 induced increased immobility time in FST was blocked by only two injections (at day 1 and 10) of a long acting KOR antagonist *nor*BNI (10 mg/kg; *i.p*.) and daily coadministration of a tricyclic antidepressant imipramine (10 mg/kg; *20 days*), but not by two SSRIs, fluoxetine (10 mg/kg, 20 days) and citalopram (10 mg/kg, 20 days) (Fig. 1A,B). Since several groups have demonstrated the given dose of fluoxetine and citalopram (10 mg/kg) to be effective in various paradigms of depression^24^,^25^, we determined the effect of these SSRIs in other paradigms of depressive behaviors. Anhedonia, the inability to experience pleasure, has been recognized as a very common endophenotype of depression^26^, we sought to determine the effect of chronic KOR activation on sucrose preference and sociability. Similar to FST results, we found that U50488 treated animals exhibit significant decrease in the sucrose preference (*F*_3,21_ = 4.710, *p* < 0.05; one-way ANOVA), and sociability deficit in social interaction test (*F*_7,122_ = 13.96, *p* < 0.0001; one-way ANOVA), which was blocked by *nor*BNI and imipramine, but not by fluoxetine and citalopram (Fig. 1C,D). Since, SSRIs were not effective in chronic U50488 induced depressive behavior, we further evaluated the effect of fluoxetine and citalopram in chronic unpredictable stress (CUS) induced depression like behaviors in C57BL/6J mice to confirm if these two drugs are fine. As expected we found that both SSRIs significantly blocked the effect of CUS in FST, SPT and sociability test (Supplementary Fig. S2A–C).

### Chronic KOR activation modulates NMDA

**Figure 2:**
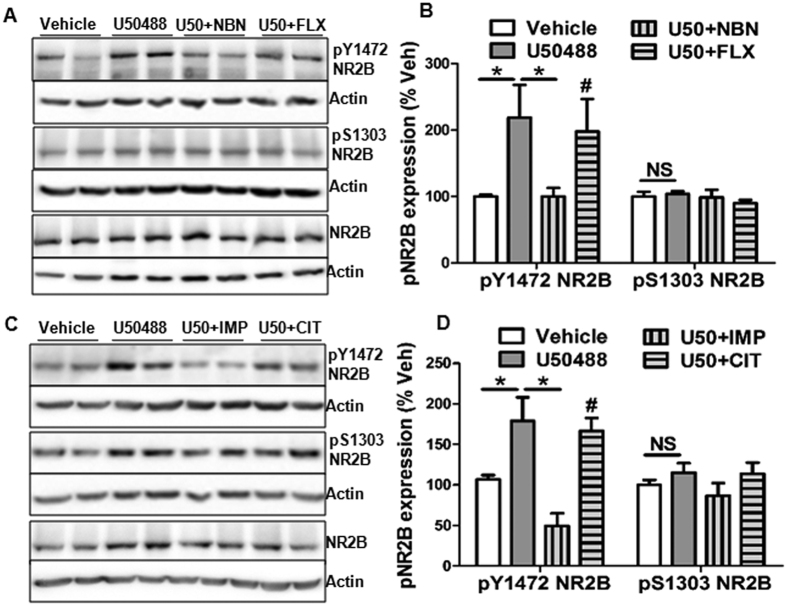
U50488 treatment increases phosphorylation of NR2B subunit of NMDA (pY1472 NMDA) in the hippocampus. (**A**) Representative western blot analysis showing increased phosphorylated NR2B (pY1472 NR2B). (**B**) Quantitative analysis of bands of pY1472 NR2B normalized to NR2B and presented as percent vehicle (%veh). *p < 0.05, student’s t-test, n = 6–7/group. (**C**) Representative western blot analysis showing the reversal of U50488 induced increased pY1472 NR2B with imipramine treatment, but not by citalopram. (**D**) Densitometry of immunoblots of pY1472 NR2B normalized to NR2B. *p < 0.05, one-way ANOVA followed by Newman-Keuls *post hoc* analysis for multiple comparisons (n = 5–6/group). Each bar represents mean ± SEM. No change in the levels of pS1303 subunits of NMDA glutamate receptor by U50488 treatment. U50 = U50488, NBN = norBNI, FLX = Fluoxetine, IMP = Imipramine, CIT=Citalopram.

Since, NMDA receptor antagonists have been shown to be effective in treatment-resistant depression^27^, we sought to determine the effect of KOR activation on the levels of NR2A and NR2B in PFC and hippocampus, widely implicated brain regions in major depression. Intriguingly, we observed that in the hippocampus, but not in the PFC, chronic KOR activation significantly increased phosphorylation of NR2B subunit (pY1472 NR2B) without affecting phosphorylation at serine 1303 (Fig. 2A,B; *p* > 0.05). Further, this increased pY1472 NR2B was blocked by norBNI and imipramine (*p*< 0.05), but not by fluoxetine or citalopram (Fig. 2A–D). Furthermore, we did not find any change in the hippocampal levels of phospho-NR2A (Y1246) or total NR2A subunit (Supplementary Fig. S3A,B) and PFC levels of pY1472 NR2B level by U50488 treatment (Supplementary Fig. S3C,D). Thus, we found a direct association between KOR mediated increase in pY1472 NR2B in the hippocampus and resistance to antidepressant action of SSRIs. We also evaluated the effect of CUS on total NR2B and pY1472 NR2B expression to determine if the two conditions (KOR activation and CUS) act in the similar way or not. In accord with previous studies showing decreased NR2B levels in different brain areas^28^,^29^, we also found that CUS decreases total NR2B expression (normalized to actin), while no effect on pY1472 NR2B (normalized to NR2B) in the hippocampus (Supplementary Fig. S4A–C).

**Figure 3:**
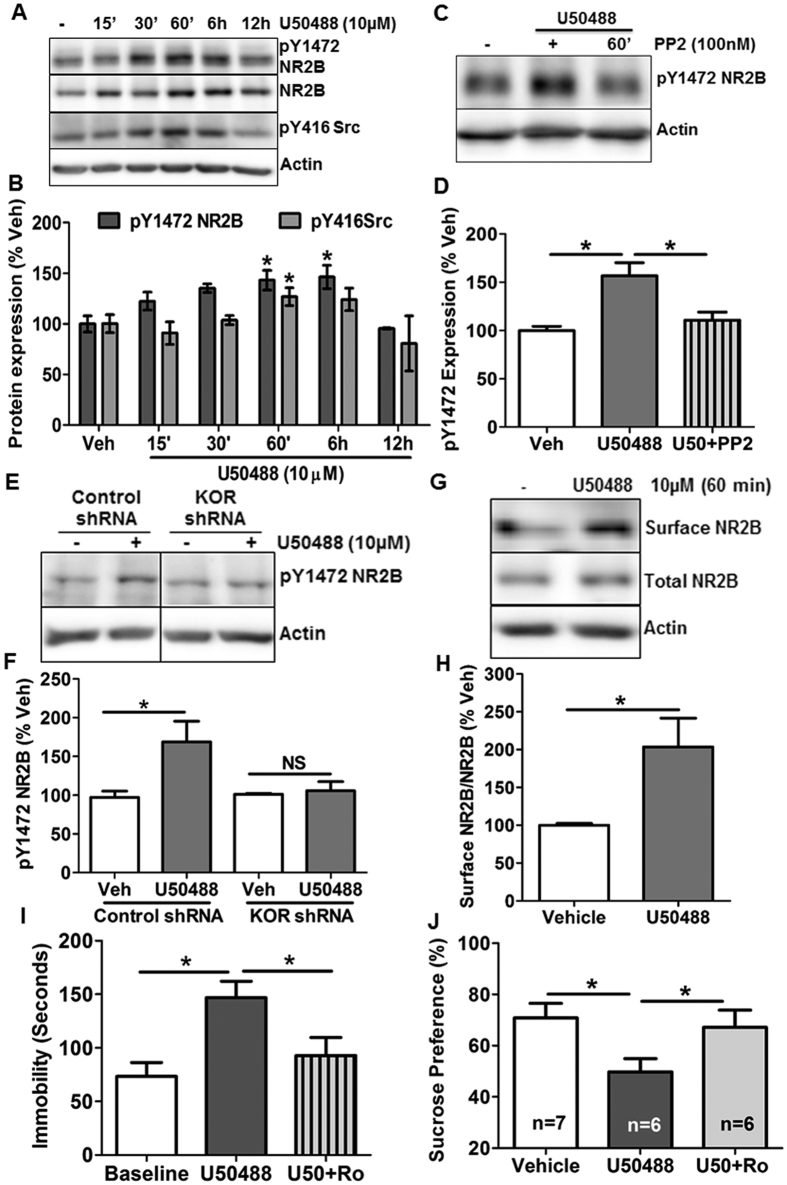
KOR modulates NR2B-NMDARs in the primary hippocampal neurons and reversal of KOR induced prodepressive behavior in the mice by NR2B NMDAR selective inhibitor Ro 25-6981. (**A**) Representative immunoblots depicting concomitant increase of pY1472 NR2B and pY416 Src kinase due to U50488 (10 μM) treatment in the primary hippocampal neurons. (**B**) Densitometry of the immunoblots of pY1472 NR2B and pY416 Src normalized to actin levels. Each bar is represented as %vehicle. *p < 0.05, one-way ANOVA followed by Newman-Keuls *post hoc* analysis for multiple comparisons, n = 5-7/group. (**C**) Representative western blot showing inhibition of pY1472 NR2B in the primary hippocampal neurons treated with Src kinase inhibitor PP2 (100 nM) thirty minutes prior to U50488 (10 μM). (**D**) Densitometry of the immunoblots (shown in C) of pY1472 NR2B normalized to actin. Data are presented as mean ± SEM of %vehicle. *p < 0.05 compared to the vehicle by one-way ANOVA followed by Newman-Keuls *post hoc* analysis, n = 5–8/group. (**E**) Representative western blot analysis showing complete blockade of U50488 (10 μM, 6 hours) induced pY1472 NR2B by shRNA-mediated knockdown of KOR in the primary hippocampal neurons. (**F**) Densitometry of the immunoblots (shown in **E**) of pY1472 NR2B normalized to actin. Data are presented as mean ± SEM of %vehicle group. *p < 0.05, n = 5–6/group. **(G)** Representative western blot showing increased surface NR2B in U50488 treated primary hippocampal neurons. **(H)** Densitometry of the immunoblots of surface NR2B normalized to total NR2B. Data are presented as mean ± SEM of %vehicle group. *p < 0.05 by student’s t-test, n = 7–9/group. (**I**) Treatment with Ro 25-6981, (10 mg/kg, *i.p*.) sixty minutes prior to FST on day 20^th^, significantly reversed immobility time equivalent to vehicle treated animals. *p < 0.05 by student’s t-test, n = 9/group. (**J**) Chronic treatment with U50488 significantly inhibited %sucrose preference as compared to vehicle treated animals on day 19. Ro 25-6981 (10 mg/kg, *i.p*.) administration on day 20^th^ to those animals that were given U50488 for last 19 days significantly reversed the effect of U50488 on sucrose preference. *p < 0.05 by student’s t-test, n = 6–7/group.

To further dissect the mechanism and specificity of U50488 mediated phosphorylation of NR2B in the hippocampus, we used primary hippocampal neuronal cultures. As shown in Fig. 3, U50488 treatment increased pY1472 NR2B in time dependent manner with a statistically significant increase after 1h and 6 h of U50488 treatment (Fig. 3C,D; *F*_5,13_ = 5.434, *p* < 0.05; one-way ANOVA). Dynorphin A (1–13), a peptide agonist of KOR which has been shown to have potency and selectivity as good as Dyn A (1–17)^30^,^31^, also upregulated pY1472 NR2B in a time dependent manner (Supplementary Fig. S5A,B; *F*_4,17_ = 4.42, *p* < 0.05; one-way ANOVA). Since Src protein tyrosine kinases have been reported to regulate NMDAR’s activity by phosphorylating NR2B^32^ and KOR activation leads to Src kinase activation, we first confirmed the effect of KOR agonists on Src kinase activation and found significant and time dependent increase in the phosphorylation of Src kinase at tyrosine 416 in the primary hippocampal neurons treated with U50488 (Fig. 3A–D; *p* < 0.05) as well as Dynorphin A (1-13) (Supplementary Fig. S5A,B; *p* < 0.05). Next, we sought to determine the effects of Src kinase inhibitor PP2 on pY1472 NR2B. As expected, we observed that KOR induced increase in pY1472 NR2B in the primary neurons was significantly blocked by PP2 (Fig. 3E,F; *F*_2,22_ = 9.731; *p* < 0.001). As reported previously^33^, we also found that NR2B phosphorylation at Y1472 causes increased surface level of NR2B in the primary hippocampal neurons (Fig. 3G,H; t(14) = 3.121, *p* < 0.01). Considering the promiscuous nature of most of the kinase inhibitors, we further confirmed the specificity of cross talk between KOR and NMDA using KOR shRNA-mediated knockdown of KOR in the primary neurons (Supplementary Fig. S6A,B). The results of this experiment clearly showed that KOR mediated (U50488 treatment for 1 hr) increase in pY1472 NR2B was completely abrogated by the knockdown of KOR in the primary neurons (Fig. 3G,H; *F*_3,15_ = 4.913, *p* < 0.05; one-way ANOVA). These results for the first time demonstrated that KOR activation selectively modulates NR2B NMDA signaling in the hippocampal neurons.

### NR2B selective antagonist Ro 25-6981 reverses the KOR induced depression like symptoms

In view of our unambiguous results of KOR induced upregulation of pY1472 NR2B in the hippocampus and primary neurons, we investigated the effect of Ro 25-6981, a NR2B subunit selective NMDA receptor antagonist, on KOR induced depressive behaviours in mice. We found that only one dose of Ro 25-6981 (10 mg/kg, *i.p*.) significantly reversed the U50488 induced immobility in FST (Fig. 3I; t(8) = 3.8, *p* < 0.01) and sucrose preference (Fig. 3J; t(5) = 2.882, *p* < 0.05). Notably, other groups have also shown the antidepressant effects, without any psychomotor effect, of Ro 25-6981 at same dose in other mouse model of depression^34^,^35^. These findings, together with the observed differential phosphorylation of the NR2A and NR2B subunits due to sustained KOR activity, suggest an essential role of NMDARs signaling in KOR mediated depression like behaviors in the mice. Furthermore, we also determined if norBNI acts as a fast acting antidepressant (*i.p.*, 10 mg/kg for 60 min) in CUS, but we did not observe any effect of norBNI on immobility time measured by FST in CUS treated animals (Supplementary Fig. S7).

### Chronic U50488 treatment alters Rac1 expression in hippocampus

**Figure 4:**
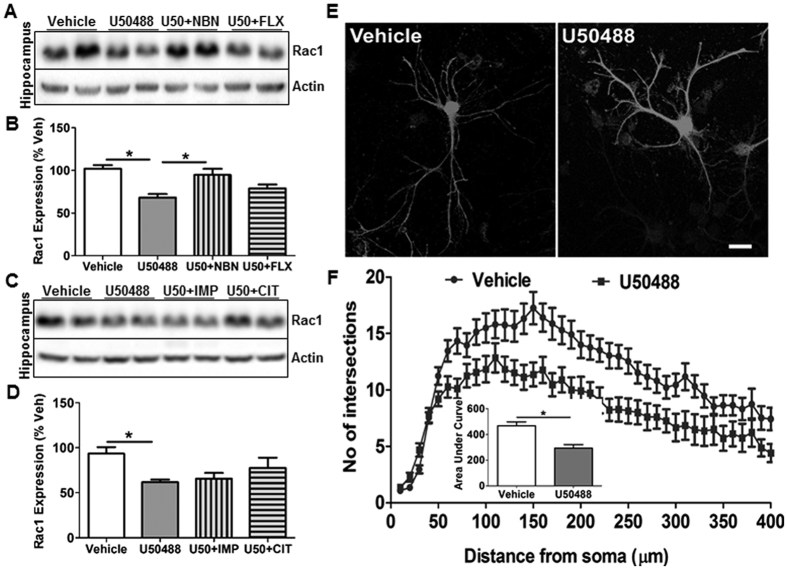
KOR modulates Rac1 expression in the hippocampus. (**A,C**) Representative immunoblot of Rac1 expression in the hippocampus showing significantly suppressed Rac1 expression by U50488 treatment and its blockade by norBNI (10 mg/kg), but not by citalopram, fluoxetine or imipramine cotreatment. (**B,D**) Densitometry of the immunoblots of Rac1 normalized to actin. Each bar represents mean ± SEM of %vehicle. *p < 0.05, one way ANOVA followed by Newman-Keuls *post hoc* analysis, n = 6-8/group. Each bar represents mean ± SEM of %vehicle. **(E,F)** U50488 treatment (10 μM, 48h) decreased dendritic arborization in the primary hippocampal neurons. (Inset) Histogram showing decreased area under the sholl analysis curve in the U50488 treated neurons, *p < 0.0001 by student’s t-test, n = 40–50 neurons/group from two independent experiments.

Impaired Rac1 function in dendritic arborization and spines have been reported in the post-mortem brain of MDD patients, and rodent models of depression^36^. Previous studies have also shown that NMDA receptors indirectly regulate activation of Rac1 to control neuronal morphology^37^,^38^. Therefore, we hypothesized that KOR induced NR2B signaling might perturb Rac1 expression and/or activity in this model of depression. As anticipated, we found that U50488 treatment significantly decreased Rac1 expression in the hippocampus (*F*_3,34_ = 10.13, *p* < 0.0001), which was significantly attenuated by norBNI (*p* < 0.05) only, but not by fluoxetine, imipramine or citalopram (Fig. 4A–D). However, chronic KOR activation did not change Rac1 expression in the PFC (Supplementary Fig. S8A,B; *p* > 0.05). Furthermore, we determined the effect of U50488 on dendritic arborization in the primary hippocampal neurons and found that U50488 treated neurons exhibited significantly suppressed dendritic arbor (t(87) = 4.578, *p* < 0.0001) as determined by sholl analysis (Fig. 4E,F).

### KOR activation causes downregulation of BDNF in cortex and hippocampus

**Figure 5:**
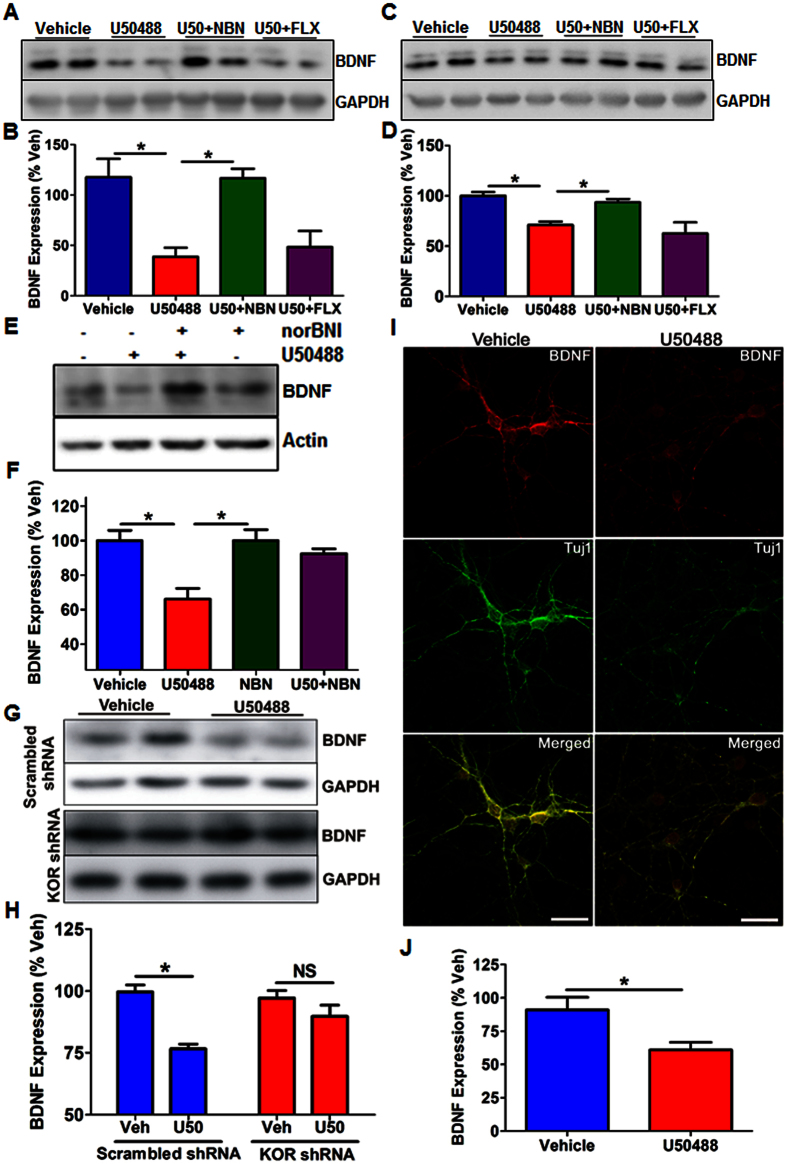
KOR regulates BDNF expression in PFC, hippocampus and primary cortical neurons. (**A**) Representative immunoblot of BDNF expression level in the cytosolic fractions of PFC. U50488 treatment significantly decreased BDNF in the PFC, which was blocked by norBNI treatment, but not by fluoxetine treatment. (**B**) Densitometry analysis of BDNF expression (normalized to GAPDH) in the PFC, each bar represents mean ± SEM of % vehicle. *p < 0.01, one way ANOVA followed by Newman-Keuls *post hoc* analysis (n = 5–7/group). (**C**) Representative immunoblot showing BDNF expression levels in the cytosolic fractions of the hippocampi. KOR activation significantly suppressed BDNF expression in the hippocampus, which were blocked by norBNI, but not by fluoxetine. (**D**) Densitometry analysis of BDNF expression (normalized to GAPDH) in the hippocampus. Each bar represents mean ± SEM of % vehicle. *p < 0.01, one way ANOVA followed by Newman-Keuls *post hoc* analysis (n = 5–7/group). (**E,F**) Representative immunoblot and its densitometry showing reduced BDNF expression in 12 days old (DIV12) primary cortical neurons treated with U50488 (1 μM, 48 hours). Each bar represents BDNF level (normalized to GAPDH) as mean ± SEM of %vehicle. *p <  0.01, one way ANOVA followed Newman-Keuls *post hoc* analysis (n = 4–6/group). (**G**) shRNA mediated knockdown of KOR in the primary cortical neurons significantly attenuated the effect of U50488 on BDNF expression. (**H**) Densitometry of the immunoblots of BDNF (shown in figure G) normalized to GAPDH. Each bar represents BDNF level as mean ± SEM of %vehicle (n = 4–5/group), *p < 0.001, one way ANOVA followed by Newman-Keuls *post hoc* analysis. (**I**) Representative confocal images of cortical neurons treated with U50488 (1 μM, 48 hours). Cortical neurons were stained for BDNF (Red) and β-3 tubulin (Tuj1, green). (**J**) Quantification of pixel intensity of BDNF immunoreactivity normalized to Tuj1 immunoreactivity per field.*p < 0.05 by student’s t-test, n = 6/group. Scale bar = 25 μm.

Since BDNF has been shown to play critical role in antidepressant response^39^, we investigated the effect of KOR activation on BDNF levels in the PFC and hippocampus. We found that U50488 treatment significantly decreased BDNF expression in the hippocampus (Fig. 5C,D; *F*_3,16_ = 8.64, *p* < 0.01) as well as PFC (Fig. 5A,B; *F*_3,9_  = 8.64, *p* < 0.01). Interestingly, this decreased BDNF levels in the hippocampus were occluded by norBNI (*p* < 0.05) and citalopram (*p* < 0.05), but not significantly by fluoxetine or imipramine treatment (Fig. 5C,D and Supplementary Fig. S9A,B). Unexpectedly, BDNF levels in the PFC were normalized by norBNI as well as imipramine (*p* < 0.05), but not by fluoxetine or citalopram (Fig. 5A,B and Supplementary Fig. S9C,D).

To further investigate if *in vivo* suppression of BDNF expression by U50488 was due to direct effect of KOR activation on neurons, we determined BDNF expression in primary cortical neurons treated with U50488 (1 μM, 48 hours) either alone or along with norBNI (10 μM, 48 hours) at DIV 12 by western blotting. We found that BDNF expression was significantly decreased in U50488 treated neurons as compare to vehicle treated neurons (Fig. 5E,F; *F*_3,17_ = 8.499, *p* < 0.01). This U50488 mediated decrease in BDNF was significantly blocked by norBNI pre-treatment (Fig. 5E,F; *p* < 0.01), suggesting the direct modulation of BDNF expression by KOR. Also, we observed decreased expression of BDNF in U50488 treated groups in immunostaining experiments (Fig. 5I,J and Supplementary Fig. S10). To further determine the specificity of U50488 on BDNF expression, we also knocked down KOR expression in the neurons by KOR specific shRNA lentiviruses for 72 hrs. As expected, we observed that U50488 mediated decrease of BDNF expression was completely abolished in KOR shRNA transduced neurons, while no significant effect in scrambled shRNA transduced neurons (Fig. 5G,H; *F*_3,14_ = 14.07; *p* < 0.001).

## Discussion

It has long been understood that acute KOR activation leads to psychotomimetic and depression like symptoms due to inhibition of monoaminergic system in various brain regions^40^,^41^. Moreover, recent findings from the ongoing clinical trials with KOR antagonists suggest that KOR antagonists exhibit beneficial effects in patients with refractory depression^42^,^43^. This implies that the blockade of KOR signaling could be rebalancing one or more unique pathways and systems that are perturbed in treatment resistant patients and can’t be influenced by SSRIs. In this study, first we modelled the treatment resistant depression by chronically activating KOR in mice with selective agonist and showed that depression like symptoms (behavioral despair, and anhedonia and sociability deficit) were blocked by norBNI and imipramine, but not by widely prescribed SSRIs fluoxetine or citalopram. Significantly, a search for the molecular determinant of this refractory depression revealed that NR2B-NMDARs in the hippocampus, but not in the PFC, are involved in KOR mediated depression. We for the first time report that KOR activation causes changes in BDNF and Rac1 expression, which are considered as neurobiological markers of depression, but hippocampal level of these markers do not match with behavioral response to antidepressants in this model.

Given that SSRIs were ineffective in correcting depression related behaviors in our model, and NMDAR antagonists are effective in the treatment resistant depression^10^,^27^,^44^, we presumed that abnormal glutamatergic signaling might be playing a role in KOR induced refractory depression. Remarkably, NR2B selective antagonist Ro 25-6981 significantly reversed the KOR induced depression like behaviors, supporting our view of KOR-NMDA cross talk in refractory depression. Furthermore, our observation of KOR mediated enhanced phospho-NR2B and cell surface NR2B in cultured hippocampal neurons elucidates the mechanisms of cross-talk between KOR and NMDARs. Since KOR has been found to be enriched in somatostatin expressing GABA interneurons in the CA1 regions of the hippocampus^45^ and our observation of KOR mediated increased NR2B signaling, it is quite plausible that persistent KOR activation overall suppresses pyramidal neurons’ activity in the hippocampus, and consequently prodepressive behaviors in the mice.

Rac1 is a member of the Rho family of GTPases, which is widely known to regulate a diverse array of cellular events, including the control of cell growth, cytoskeletal reorganization, and the activation of protein kinases^46^. Abnormal dendritic spine density and morphology due to altered Rac1 activity have been reported in several mental disorders, including depression^36^,^47^. In agreement to these studies in various rodent models of “Major Depression”, we also observed a significant decrease in Rac1 expression in the hippocampus, while no change in the frontal cortex. This dichotomy in KOR action on Rac1 between the hippocampus and frontal cortex might be due to KOR being differentially targeted in frontal cortex and hippocampus^45^,^48^. Although NR2B NMDAR signaling has been shown to positively correlate with Rac1 activity^49^, our results indicate an inverse relationship between these two proteins. Since KOR is expressed mostly in the GABAergic interneurons of hippocampus^45^, and Rac1 modulates synaptic architecture in the glutamatergic neurons^50^, it is quite possible that KOR mediated increase in NR2B-NMDARs and decrease in Rac1 expression are occurring in two different population of neurons. Since imipramine was unable to revert U50488 mediated decrease in hippocampal Rac1 expression, it seems that restoration of Rac1 expression is not necessary for the antidepressant response in this model.

The “neurotrophin hypothesis of depression”, a prevailing concept in the field of depression is based on mostly an inverse relationship between BDNF levels in the hippocampus and depression. During past two decades, numerous preclinical^51^,^52^ and clinical evidences^39^,^53^ strongly support the important role of BDNF in antidepressant action. However, several studies deviated from the neurotrophin hypothesis and showed either no change in BDNF expression with chronic fluoxetine^54^ or even opposite effects of BDNF^55^. Thus, SSRIs’ effect is not consistent in all the models of depression. In the same line, our observation of fluoxetine and citalopram not being effective in blocking prodepressive behaviors induced by persistent KOR activation lead us to postulate that SSRIs may not normalize BDNF levels in all brain regions adequately, and therefore can’t ameliorate whole range of depressive behaviors that result due to the functional integration of KOR expressing neurocircuits spanning several brain regions. In fact, we did find that citalopram significantly mitigates BDNF in the hippocampus but not in the frontal cortex, while fluoxetine does not change BNDF in both regions. Thus, it appears that restoration of BDNF levels in the hippocampus was not necessary for the antidepressant effect in this model, as citalopram restored it, but not imipramine. Importantly, BDNF expression in PFC is consistent with the effect of norBNI and imipramine, suggesting that restoration of BDNF in PFC is essential for antidepressant efficacy.

Notably, our results of mice and primary neuronal experiments unambiguously demonstrated the direct inhibitory effect of KOR signaling on BDNF expression, corroborating other sudies^22^ for the role of KOR in depression like behaviors. Based upon the data presented in this study, we propose that persistent activation of KOR causes increased NMDAR signaling via Src kinases in the hippocampus, and consequently treatment resistant depression like symptoms in mice. These findings unexpectedly reveal the essential relationship between two clinically very relevant pathways that are being pursued for the development of better therapeutics for depression. Although lack of antidepressant response of two SSRIs evaluated in this study suggest the activation of KOR in treatment resistant depression, further investigation with selective norepinephrine reuptake inhibitors and other serotonerigc antidepressants are warranted to comprehend the underlying molecular pathways completely.

## Material and Methods

### Animals

All *in vivo* experiments and procedures were performed in accordance with the guidelines established in the guide for the care and use of laboratory animals and were approved by the institutional animal ethics committee (IAEC) of CSIR-Central Drug Research Institute, Lucknow, India. The IAEC is certified by animal welfare board of India (AWBI) and committee for the purpose of supervision and control of experiments on animals (CPCSEA), which are statutory bodies of Government of India. Male C57BL/6J mice (6–8 weeks old) weighing 22–25 g were used in this study. Animals were housed on a 12- h light/dark cycle (lights on at 8.00 am). Food and water were provided *ad libitum*.

### Drugs

(−)U50488 hydrochloride, nor-Binaltorphimine dihydrochloride (norBNI), imipramine, fluoxetine, PP2 and Ro 25-6981 were purchased from Sigma-Aldrich. For experiments in mice, all drugs (except Ro 25-6981) were dissolved initially in 100% DMSO and further diluted with 0.8% sodium chloride to final 2.5% of DMSO. All drugs were administered via *intra peritoneal* (*i.p.*) injections. For *in vitro* experiments, the drugs were diluted in culture media. In all experiments, appropriate vehicle controls were used.

### Behavioral measurements

Each behavioral task involving C57BL/6J mice and biochemical assays from mice tissues in this study were conducted in accordance with guidelines of IAEC and approved by CSIR-Central Drug Research Institute, Lucknow, India. The IAEC is certified by animal welfare board of India (AWBI) and committee for the purpose of supervision and control of experiments on animals (CPCSEA), which are statutory bodies of Government of India. All behavioral experiments, except with Ro 25-6981, were performed only after 14–16 hrs of the drug treatment during 11 AM to 3 PM.

#### Forced Swim Test (FST)

To determine the effect of various drug treatments in forced swim test (FST, a measure of behavioral despair), immobility time was scored before the start of the drug administration (day-0) and last day (day-20) of the treatment. Briefly, animals were put in the cylinder filled with water (temperature 25 ± 1 °C) for five minutes and immobility time during this whole time was determined by online analysis of videos using AnyMaze 4.7 software (Stoelting, USA).

#### Social behavior testing

Sociability of the animals was tested using a 3-chambered social behavior test box as described previously^56^. Briefly, the mouse was allowed to explore the whole chamber for 10 minutes (acclimatization period). Following this acclimatization phase, an unfamiliar mouse (C57BL/6J, stranger) was placed in the wired cage kept in one of the side chamber (social chamber). Test mouse was place in the middle chamber and was allowed to explore the entire box for 10 minutes. The mouse activity was recorded by a camera and the time spent in each chamber was analyzed by AnyMaze 4.7 software (Stoelting, USA).

#### Sucrose Preference Test (SPT)

To determine the sucrose preference, mice were trained with two bottles, one with 1% sucrose solution (Sigma-Aldrich) and another with water for 48 hours. To circumvent the possible effects of side preference, the position of bottles was switched after 12 hour. Training was followed by a five-hour of water deprivation and a two-hour testing period with preweighed two identical bottles, 1% sucrose solution and water. Water and sucrose solution consumption was calculated by measuring the change in the weight of fluid consumed. Sucrose preference was calculated as the ratio of the weight of sucrose solution consumed versus total liquid consumption during testing period.

### Primary neuronal culture and shRNA mediated knockdown

Primary neurons were cultured from the cerebral cortices and hippocampi of 0–1 day old mouse pups as described elsewhere^57^. Briefly, the cortices or hippocampi were gently dissociated in 1X HBSS (Sigma-Aldrich) and were digested in papain (0.1%) at 37 °C for 20 minutes. After digestion, tissue samples were triturated 10–15 times to get the dissociated cells, which were suspended in the neurobasal media supplemented with 2% B27 supplement (Invitrogen, #17504) and 0.5 mM glutamax (Gibco, #35050) onto 12-well tissue culture plate (0.5 × 10^6^ cells/well) coated with poly-L-lysine (0.1 mg/ml) and incubated at 37 °C in a humidified atmosphere of 95% air and 5% CO_2_. Lentiviruses containing KOR shRNA (NM_011011, Sigma Aldrich) were packaged using pMDG.2 (Addgene; #12259) and psPAX2 (Addgene; #12260) as packaging plasmids in HEK293T cell line as described elsewhere^58^. To knockdown KOR from primary neurons, the viral transduction was done at days *in vitro* 8–9 (DIV 8–9) and all drug treatments were done after 48 hours of transduction, and neuronal cell lysates was prepared by adding RIPA buffer with protease inhibitor cocktails and phosphatase inhibitor cocktails to the neurons directly.

### Adeno associated virus (AAV) packaging and determination of dendritic arbor

AAV2 was packaged in HEK293 cell line as described elsewhere^59^. Briefly, pTR-CBA-TdTomato, pXX6-80 and pXR2 (These plasmids were kind gift from Dr. Aravind Asokan, UNC- Chapel Hill, USA) were transfected in HEK293 cell line and AAV2 particles were purified using a discontinuous iodixanol gradient (Sigma-Aldrich). For primary neuronal transduction (DIV5), 1 μl of AAV2 viral particles (1 × 10^10^ copies/ml) containing pTR-CBA-TdTomato was added to the neuronal culture. U50488 (10 μM) was added to the neurons after 24 h of viral transduction and were fixed with 4% PFA (pH7.2) after 48 h of U50488 treatment. The fixed neurons were stained with anti-RFP and were imaged.

### Brain tissue isolation and Western Blotting

The mice were euthanized after overdose of anaesthetics (350 mg/kg of avertin) by decapitation and PFC and hippocampi were rapidly microdissected, snap-frozen in liquid nitrogen and stored at −80 °C until usage. Western blotting of protein from the brain tissue samples and primary neurons was done as described elsewhere^60^. Briefly, 40–50 μg of protein samples were subjected to 8–12% SDS-PAGE and elctro-transferred to polyvinyl difluoride (PVDF) membrane. After at least two hours of blocking at room temperature, specific primary antibodies (Supplementary Table S1 for details of each antibodies used in this study) were added for overnight at 4 °C followed by incubation with horseradish peroxidase conjugated secondary antibodies. The immunoreactive bands of various proteins were visualized using enhanced chemiluminescence solution (Merck-Millipore, India) and gel documentation system (MyECL Imager, Thermo Scientific, USA). All quantifications were performed with MyImage analysis software (Thermo Scientific, USA).

### Measurement of cell surface NR2B

The plasma membrane level of NR2B in cultured hippocampal neurons were determined by cell surface biotinylation using Sulfo-NHS-SS-Biotin (Thermo-scientific, USA) following manufacturer’s instructions. Biotin labelled cell lysate was immunoprecipitated using NeutrAvidin agarose resin and immunoblotted by NR2B specific primary antibody.

### Immunocytochemistry and confocal imaging

For primary neurons staining, PFA fixed neurons were blocked with blocking buffer containing 3% BSA, 3% horse serum, 0.3% Triton X100 (in 1X PBS) for two hours at room temperature followed by incubation with primary antibodies Supplementary Table S1) for 48 h at 4 °C and species specific AlexaFluor-488, −594 (1:1000; Molecular Probes) tagged secondary antibodies for one hour at room temperature. Fluorescence signals were captured under Leica SP8 confocal microscope equipped with high sensitivity Hyd detectors using 40X (0.85NA) PlanApo objective. Images were collected with xyz acquisition mode with the 1 μm step size where the total thickness of sample ranged between 13–15 μm. All the optical sections were projected as a single image for presentation. Quantification of fluorescence signals was done by drawing region of Interest on the full frame and arbitrary fluorescence units (grey values) were normalized using Tuj1 signals in each image. To study dendritic arborization, anti-RFP stained neurons were imaged using Olympus BX61-FV1200-MPE microscope equipped with HSD2 detectors using 40X oil (1.3NA) objective with 4.0 μs/pixel scan speed. The confocal images were analyzed using ImageJ software (www.rcb.info.nih.gov/ij) which has Sholl analysis^61^ plugin installed. Cropped images were assembled for presentation in Adobe Photoshop (version 7.1).

### Data Analysis

Statistical analysis was performed using GraphPad Prism 5 software (La Jolla, California, USA). Behavioral data from forced swim test was analyzed using two-way analysis of variance followed by Bonferroni *post hoc* test to determine behavioral changes for pre-treatment and post treatment. Effect of various treatments on protein expressions were analyzed by one-way ANOVA followed by Newman-Keuls *post hoc* test.

## Additional Information

**How to cite this article**: Dogra, S. *et al*. Chronic Kappa opioid receptor activation modulates NR2B: Implication in treatment resistant depression. *Sci. Rep.* **6**, 33401; doi: 10.1038/srep33401 (2016).

## Supplementary Material

Supplementary Information
