# Supplementary material for: Chronic Kappa opioid receptor activation modulates NR2B: Implication in treatment resistant depression
Source: Sci Rep. 2016 Sep 16;6:33401. doi: 10.1038/srep33401 (PMC5025743; doi:10.1038/srep33401)
Supplement: Supplementary Information [file srep33401-s1.pdf]

Chronic Kappa opioid receptor activation modulates NR2B: Implication in treatment resistant depression

ShaliniDogra[1], Ajeet Kumar[1], Deepmala Umrao[1], Amogh A. Sahasrabuddhe[2], Prem N Yadav[1]*

[1]Division of Pharmacology,[2]Division of molecular and structural biology, CSIR-Central Drug Research Institute, Lucknow, UP 226031, India

Supplementary Figure-1.

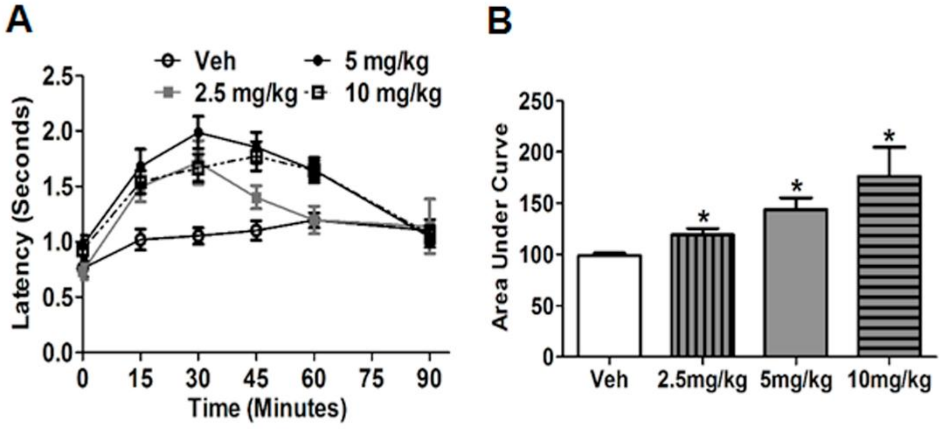

Figure S1: Analgesic effect of systemic acute treatment with U50488. **(A)** Tail Flick testing using different doses of U50488 (2.5mg/kg, 5mg/kg and 10mg/kg; *i.p.*) to find out the optimal analgesic dose of U50488. **(B)** Bar graph showing area under the curve of tail flick test graph.*p<.01 by unpaired student's t-test (n=4-6 mice/group).

Supplementary Figure-2.

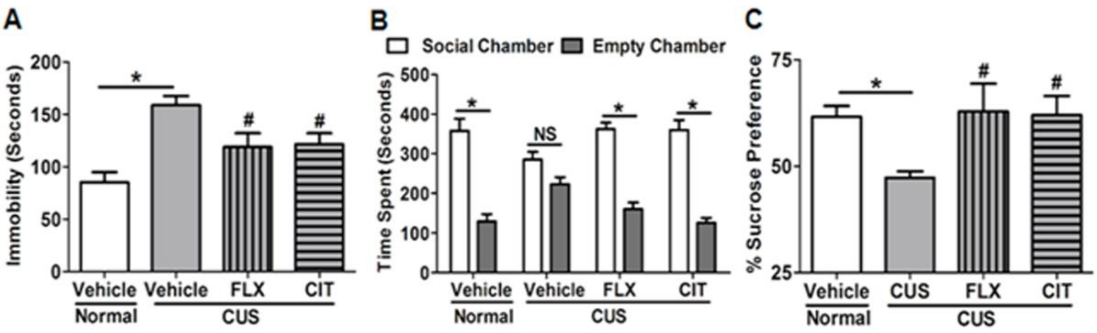

Figure S2. Reversal of CUS induced prodepressive behavior in mice by fluoxetine and citalopram. (**A**) Chronic unpredictable stress for 21 days increases immobility time in the forced swim stress test as compare to normal animals. Treatment of depressed animals (after 21 days of CUS paradigm) with fluoxetine (10mg/kg; *i.p.*) and citalopram (10mg/kg; *i.p.*) for twenty days decreased the immobility time as compared to vehicle treated depressed animals.Each histogram represents mean ± SEM of 6-8 mice/group, **p*<.001 by one-way ANOVA followed by Newman-Keuls *post hoc* analysis for multiple comparisons. **B**) CUS treated animal exhibited marked social deficit by spending significantly more time in the empty chamber as compared to normal vehicle treated animals.CUS induced impaired sociability was significantly reverted by 21 days of treatment with fluoxetine (10mg/kg; *i.p.*) and citalopram (10mg/kg; *i.p.*). Each histogram represents mean ± SEM of 6-8 mice/group, **p*<.0001 by one-way ANOVA followed by Newman-Keuls *post hoc* analysis for multiple comparisons. *comparison between an empty chamber and social chamber of each group.(C) CUS treatment significantly inhibited the percent sucrose preference as compared to normal vehicle treated animals. Twenty days of treatment with fluoxetine and citalopram reversed the decreased sucrose preferenceof CUS induced depressed mice. **p*<.05 by one-way ANOVA followed by Newman-Keuls *post hoc* analysis for multiple comparisons, n=6-8 mice/group. For **(A, C)** #comparison of fluoxetine and citalopram treated animals with CUS vehicle group. U50=U50488, FLX=Fluoxetine, CIT=Citalopram.

Supplementary Figure-3.

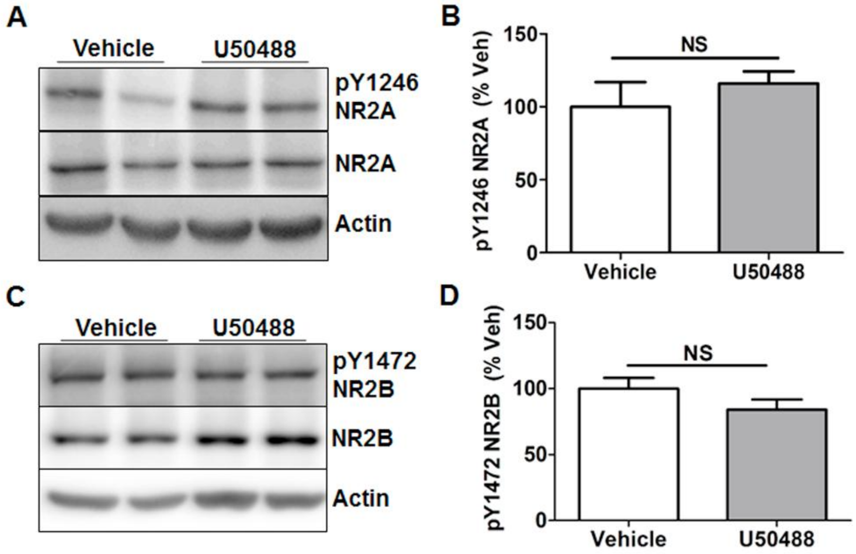

Figure S3: No effect of chronic U50488 treatment on pY1246 NR2A levels in the hippocampus and pY1472 NR2B levels in frontal cortex. **(A)** Representative western blot analysis showing no change in the levels of phosphorylated NR2A (Y1246) in U50488 treated animals. **(B)** Densitometry ofpY1246 NR2A specific band normalized to total NR2A and presented as percent vehicle (%veh), $p > .05$, student's t-test, n=5-6/group. **(C)** Representative western blot analysis showing no change in the levels of pY1472 NR2B in frontal cortex of animals treated with U50488 ($p > .05$) **(D)** Densitometry of the immunoblot of pY1472 NR2B normalized to NR2B, $p > .05$, student's t-test, n=5-6/group. Each bar represents mean ± SEM.

Supplementary Figure-4.

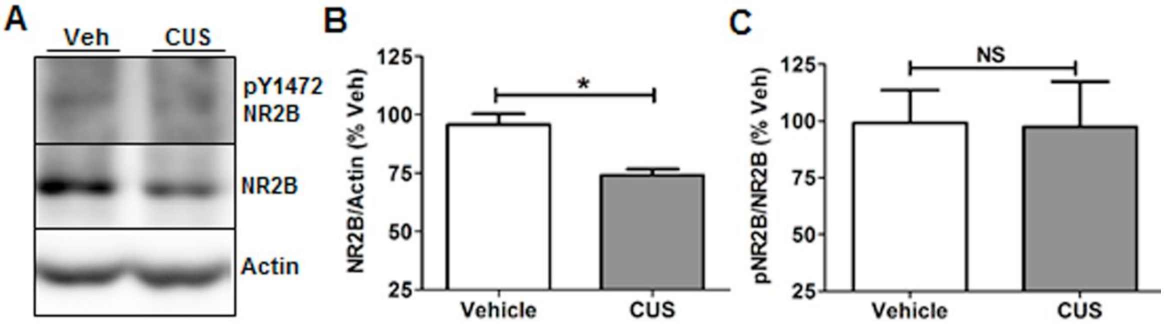

Figure S4. Chronic unpredictable stress decreases NR2B subunit of NMDA in the hippocampus. (A) Representative western blot analysis showing decreased levels of NR2B in CUS treated animals. (B) Quantitative analysis of bands of NR2B normalized to actin and presented as percent vehicle (%veh). **p*< .005, student's t-test, n=5-6/group. (C) Quantitative analysis of bands of pY1472 NR2B normalized to NR2B and presented as percent vehicle (%veh) (n=6/group). Veh=vehicle, CUS= chronic unpredictable stress.

Supplementary Figure-5.

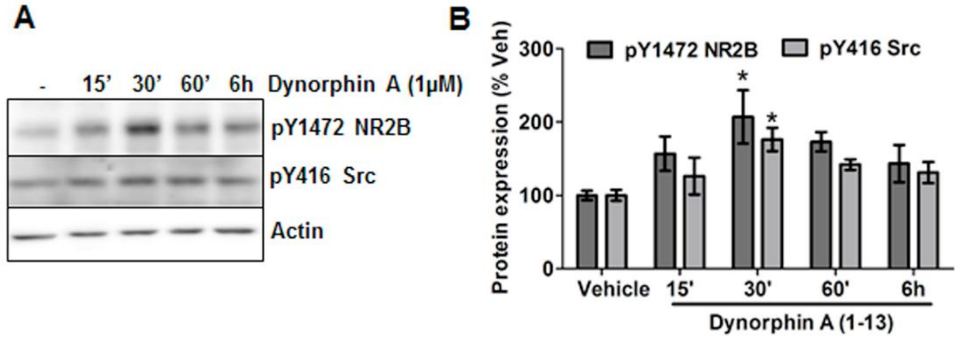

Figure S5: Dynorphin A treatment increases pY1472 NR2B and pY1472 Src in primary hippocampal neurons**. (A)** Western blot analysis of the primary hippocampal neurons treated with Dynorphin A (1-13)(1µM) after 15, 30, 60 minutes and 6hours of treatment. Dynorphin A (1-13) treatment increases pY1472 NR2B and pY416 Src kinase in the primary hippocampal neurons. **(B)** Densitometry of pY1472 NR2B and pY416Src normalized to actin. Each bar represents here as %vehicle. **p*<.05, one-way ANOVA followed by Newman-Keuls *post hoc* analysis for multiple comparisons, n=4-5/group.

Supplementary Figure-6.

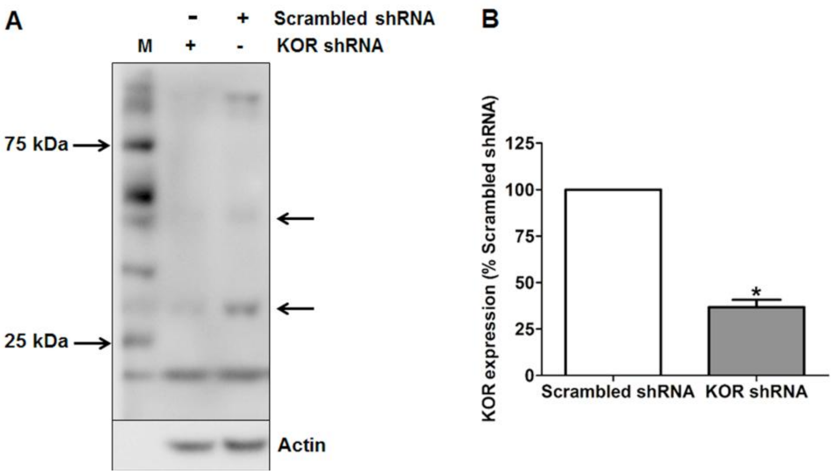

Figure S6: KOR shRNA treatment decreases KOR expression in the primary neurons. **(A,B)** Representative western blot and densitometry analysis showing decreased KOR expression after shRNA mediated knockdown of KOR in the primary neurons. For shRNA mediated knockdown, scrambled and KOR shRNAs packaged in lentiviruses were added to the neurons at DIV8-9. Seventy two hours post transduction, neurons were harvested and protein was subjected to WGA pulldown. Total eluate of WGA pulldown were immunoblotted using KOR specific antibody.

Supplementary Figure-7.

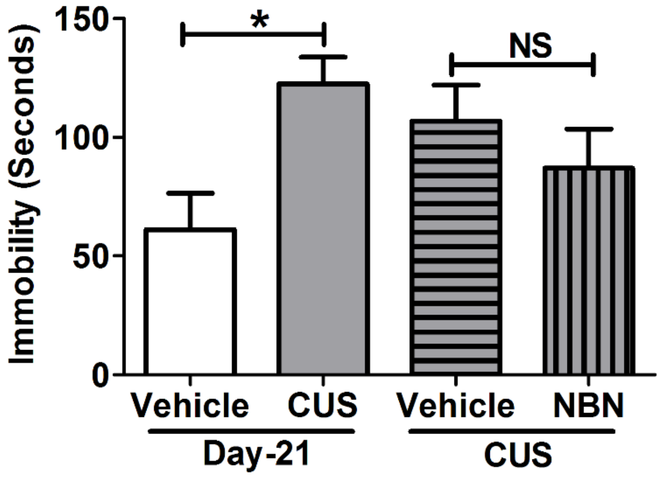

Figure S7. Acute effect of norBNI in CUS treated animals. (A) Chronic unpredictable stress for 21 days increases immobility time in the forced swim test as compared to normal animals. Treatment of depressed animals (after 21 days of CUS paradigm) with norBNI (10 mg/kg; *i.p.*) for sixty minutes prior to FST does not affect the immobility time as compared to vehicle treated depressed animals. Each histogram represents mean ± SEM of 6-8 mice/group, **p* < .05 by student's t-test, NS=non significant.

Supplementary Figure-8.

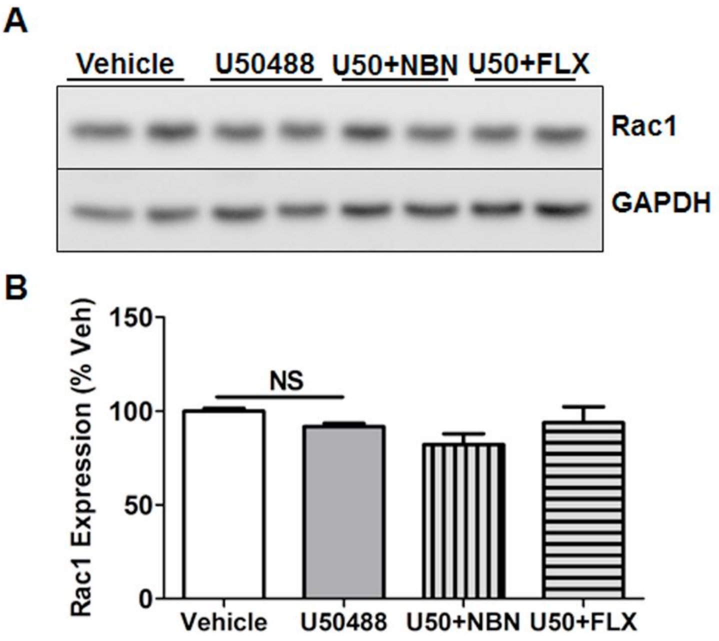

Figure S8: Chronic KOR activation doesnot affect Rac1 expression in the prefrontal cortex. **(A)** Representative immunoblot of Rac1 expression in the prefrontal cortex showing no effect of KOR activation on Rac1 expression **(B)** Densitometry of Rac1 specific bands normalized to GAPDH level. Each bar represents mean ± SEM of %vehicle. *p>.05, student's t-test, n=4-5/group.

Supplementary Figure-9.

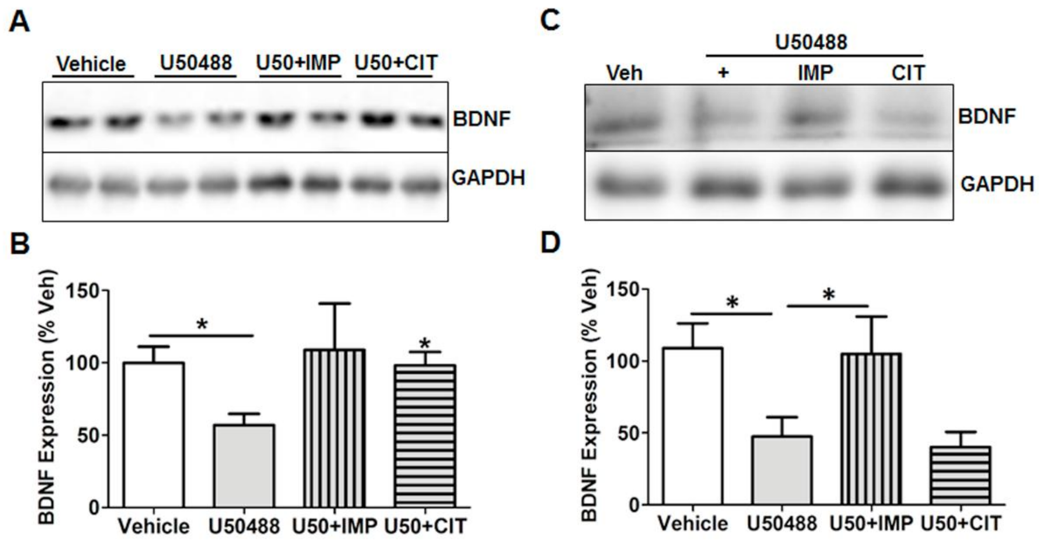

Figure S9. Effect of antidepressants on U50488 induced decrease in BDNF. **(A)** Representative immunoblot of BDNF expression level in the cytosolic fractions of hippocampus. Citalopram treatment attenuated the U50488 induced decrease in hippocampal BDNF expression. **(B)** Densitometry analysis of BDNF expression (normalized to GAPDH). Each bar represents mean ± SEM of %vehicle. *p<.05, student's t-test, n= 5-7/group. **(C)** Representative immunoblot of BDNF expression level in the cytosolic fractions of PFC. Imipramine treatment attenuated the U50488 induced decrease in prefrontal cortex BDNF (p< .05). **(D)** Densitometry analysis of BDNF expression (normalized to GAPDH). Each bar represents mean ± SEM of %vehicle. Each bar represents mean ± SEM of %vehicle. *p<.05, student's t-test, n=5-6/group.

Supplementary Figure-10.

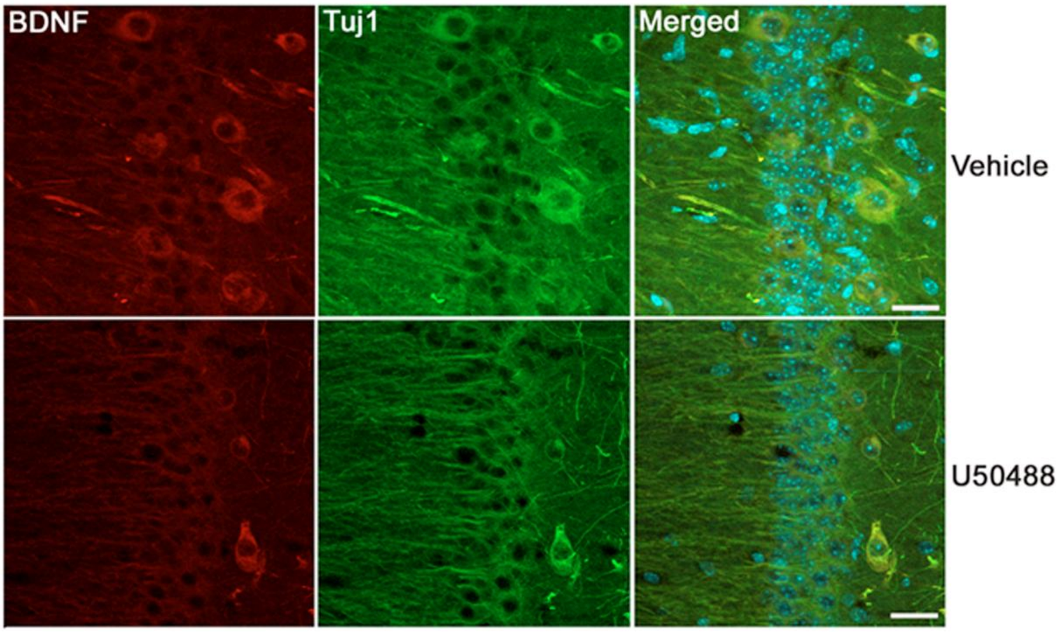

Figure S10. U50488 induced decrease in BDNF expression in the CA1 of hippocampus. A representative confocal image of CA1 region of hippocampus showing decreased BDNF immunoreactivity in the U50488 treated samples (shown in red). BDNF= brain derived neurotrophic factor, Tuj1= β-3 tubulin. Each scale bar = 20µm.

Table S1. Primary antibodies and their working concentrations.

S. No.	Antibody	Company	Catalogue Number	Working concentration
1	rabbit anti-BDNF antibody (**for western analysis**)	Santacruz Biotechnology	sc-546	1:1000
2	mouse anti-Rac1 antibody	Calbiochem	05-389	1:5000
3	rabbit anti-pS845 GluR1 antibody	Cell Signaling Technology	8084	1:2000
4	rabbit anti-pS831 GLuR1 antibody	Millipore	AB5847	1:2000
5	rabbit anti-pY1472 NR2B antibody	Abcam	ab59205	1:2000
6	rabbit anti-pS1303 NR2B antibody	Abcam	ab81271	1:2000
7	rabbit anti-NR2B antibody	Abcam	ab65783	1:4000
8	rabbit anti-p-NMDAR2A (Tyr1246) antibody	Cell Signaling technology	4206	1:1000
9	rabbit anti-NR2A antibody	Cell Signalling Technology	4205	1: 2000
10	mouse anti-Actin	Sigma Aldrich	A2228	1:10000
11	mouse anti-GAPDH	Calbiochem	CB1001	1:10000
12	sheep anti-BDNF antibody **(For IHC)**	Millipore	AB1513P	1:500
13	mouse anti-beta-3 tubulin antibody	Abcam	ab14545	1:3000
14	rabbit anti-KOR antibody	Abcam	ab10566	1:2000
15	Rabbit anti-RFP antibody	Abcam	ab62341	1:1000

Supplementary Methods:

Tail Flick Testing:

For assessment of thermal analgesia, tail flick test was done as described previously with modifications [1]. Briefly, for each testing session, mice were individually acclimatized into the restrainers for one minute without tail immersion. After acclimatization, each mouse was gently introduced into the restrainers and the distal one-third of the tail was dipped into the hot water bath (temp 52° ± 0.5°C) and tail withdrawal latencies were recorded thrice at a time point by an observer blinded to the identity of the animal.

Chronic unpredictable stress:

Chronic unpredictable stress consisted of randomized periods of 8 stressors over the course of 21 days with two stressors per day. These stressors were continuous overnight illumination (12 h), continuous daylight off (12h), wet cage (12 h; night cycle), acute restraint stress (120 min; light cycle), overcrowding (overnight), exposure to cold temperature (2h at 4°C; during light cycle), cage tilt (45°, overnight) and acute foot shock (six rounds of 3 seconds mild foot shock of intensity 0.5mA with 60 second of inter-shock interval) using GEMINI avoidance system (San Diego, USA). Mice were subjected to FST on day-0 and day-21 of CUS paradigm to measure the baseline immobility and Immobility change after CUS respectively. After FST, the mice with increased immobility time were considered as depressed. The depressed animals were treated with either normal saline (*i.p.*) or fluoxetine (10mg/kg; *i.p.*), or citalopram (10mg/kg; *i.p.*) for twenty days and social behavior, sucrose preference and FST was done on day-19, day-20 and day-21, respectively. Further, to determine the acute effect/ fast acting effect of norBNI, we injected norBNI (10mg/kg; *i.p.*) on day-22 in the previously vehicle treated CUS mice and measured immobility time in the FST after 60 minutes of norBNI administration.

Immunohistochemistry for BDNF staining:

Immunostaining of tissue sections was done as explained elsewhere [2]. Animals from each group were transcardially perfused with 4% PFA twenty four-hour after all behavioural experiments and their brains were taken out. The brains were further fixed in 4% PFA for overnight followed by dehydration in 30% sucrose solution (w/v in 1XPBS). Brains were cryosectioned at 20µm thickness using cryotome (FSE, Thermo Scientific, USA) and free floating brain sections were permeabilized with 0.5% Triton X100 followed by blocking with blocking buffer (3% BSA, 3% horse serum, 0.3% Triton X100 in 1X PBS) for two hours at room temperature. Primary antibodies were added to the blocked sections for 48 hours at 4°C (**Table S1**) followed by incubation with species specific AlexaFluor-488, -594 (1:1000; Molecular Probes) tagged secondary antibodies for two hours at room temperature. Confocal microscopy was done as described for immunocytochemistry.

Wheat Germ Agglutinin (WGA) pulldown:

To determine the degree of KOR knockdown by KOR shRNA in the primary neurons, total protein lysates were prepared using RIPA buffer after total 72 hrs of KOR shRNA or scrambled shRNA addition. Total 400 µg of precleared protein lysate was incubated with 20 µl of WGA-agarose resins and mixed by rotation at 4°C overnight before washing with lysis

buffer. Beads with attached proteins were mixed with 4X SDS-PAGE sample buffer and heated at 50°C for 5 min. Cleared elutes were collected after spinning and immunoblotted using KOR specific antibody.

References:

1 Ta, L. E., Low, P. A. & Windebank, A. J. Mice with cisplatin and oxaliplatin-induced painful neuropathy develop distinct early responses to thermal stimuli. *Mol Pain* **5**, 9 (2009).

2 Yadav, P. N. *et al.* The presynaptic component of the serotonergic system is required for clozapine's efficacy. *Neuropsychopharmacology* **36**, 638-651 (2011).
